# Characterisation of *Vibrio cholerae* isolates from the 2009, 2010 and 2016 cholera outbreaks in Lusaka province, Zambia

**DOI:** 10.11604/pamj.2020.35.32.18853

**Published:** 2020-02-07

**Authors:** Kapambwe Mwape, Geoffrey Kwenda, Annie Kalonda, John Mwaba, Chileshe Lukwesa-Musyani, Joseph Ngulube, Anthony Marius Smith, James Mwansa

**Affiliations:** 1 Department of Basic Sciences, Michael Chilufya Sata School of Medicine, Copperbelt University, Ndola, Zambia; 2 Department of Pathology and Microbiology, School of Medicine, University of Zambia, Lusaka, Zambia; 3 Department of Biomedical Sciences, School of Health Sciences, University of Zambia, Lusaka, Zambia; 4 Department of Pathology and Microbiology, University Teaching Hospital, Lusaka, Zambia; 5 Bacteriology Division, Centre for Enteric Diseases, National Institute for Communicable Diseases, National Health Laboratory Service, Johannesburg, South Africa; 6 Department of Clinical Microbiology and Infectious Diseases, School of Pathology, Faculty of Health Sciences, University of the Witwatersrand, Johannesburg, South Africa; 7 Department of Medical Microbiology, Faculty of Medicine, Lusaka Apex Medical University, Lusaka, Zambia

**Keywords:** Cholera, *Vibrio cholerae*, multidrug resistance, Lusaka province

## Abstract

**Introduction:**

In 2009 and 2010, more than 6,000 cholera cases were recorded during these outbreaks with more than 80% of cases recorded in Lusaka province. After a five-year break, in 2016 an outbreak occurred in Lusaka, causing more than 1,000 cases of cholera. This study will strengthen the epidemiological information on the changing characteristics of the cholera outbreaks, for treatment, prevention and control of the disease.

**Methods:**

This was a laboratory-based descriptive cross-sectional study conducted at the University Teaching Hospital in Lusaka, Zambia. A total of 83 *V. cholerae* O1 isolates were characterised by biochemical testing, serotyping, antimicrobial susceptibility testing, and macrorestriction analysis using Pulsed-Field Gel Electrophoresis.

**Results:**

Macrorestriction analysis of the isolates demonstrated high genetic diversity among the isolates with 16 different patterns. The largest pattern comprised 9 isolates while the smallest one had 1 isolate. 2009 and 2010 isolates were highly resistant to nalidixic acid and cotrimoxazole, but highly sensitive to azithromycin and ampicillin. Of the fifty-two isolates from the 2016 cholera outbreak, 90% (47) were sensitive to cotrimoxazole, 94% (49) to tetracycline, and 98% (51) to azithromycin, while 98% (51) were resistant to nalidixic acid and 31(60%) to ampicillin.

**Conclusion:**

macrorestriction analysis demonstrated high genetic diversity among the *V. cholerae* O1 strains, suggesting that these isolates were probably not from a similar source. This study also revealed the emergence of multidrug resistance among the 2016 *V. cholerae* outbreak isolates but were susceptible to cotrimoxazole, tetracycline, and azithromycin, which can be used for treatment of the cholera cases.

## Introduction

Cholera is an enteric disease of immense public health concern, causing an estimated 2.8 million cases with 91,000 deaths globally every year [1]. It is an acute disease characterised by severe watery diarrhoea caused by toxigenic *Vibrio cholerae* strains belonging to serogroups O1 and O139 [2]. The bacteria colonise the small intestine and produce an enterotoxin known as cholera toxin (CT) [3]. Pandemics that are caused by this bacterium have severely affected many countries on multiple continents for many years [4]. Cholera is transmitted via the faeco-oral route and is particularly associated with poverty and poor sanitation [3]. The first cholera outbreak in Africa was reported in 1836 along the Indian Ocean coast killing about 20,000 people [5]. No further outbreak was reported on the continent after the 1893-1894 outbreak in the Senegambia region until the seventh pandemic reached the continent in 1970 [6]. This pandemic caused massive outbreaks in Africa resulting in more than 400,000 cases with a high mortality rate [7]. Between 1995 and 2005, Africa experienced a greater upsurge in cholera outbreaks than other continents, with over 80% of the global total number of cholera cases [8]. The trend of Africa reporting more cholera cases continued between 2006 and 2010 [9]. In addition over the past 10 years, several Southern African countries, such as Mozambique [10], Tanzania [11], Zimbabwe [12], South Africa [2]and Zambia [13] have reported cholera outbreaks. Zambia usually experiences cholera outbreaks during the rainy season and most of them have been associated with fishing camps, especially in the northern part of the country and in unplanned settlements of Lusaka and Copperbelt Provinces [14]. The first cholera outbreak in Zambia was reported in the 1970s and several other outbreaks have occurred over the years, with the worst outbreak being in 1991 that resulted in over 13,000 cases [15]. In the 2009 outbreak, a total of 4,712 cases were reported, while the 2010 cholera outbreak caused 6,794 cases, with the majority of the cases being from Lusaka Province [13, 14]. In 2016 between February and June, Zambia experienced an outbreak with more than 1000 cases and 22 deaths being reported from Lusaka Province alone after a quiescent period of 5 years [16]. In order to minimize the disease burden caused by cholera, antimicrobial drugs have been used against *V. cholerae* O1 to shorten the duration of illness and to reduce the volume of stools produced [17]. However, the increased use of antibiotics against cholera has resulted in the emergence of multiple drug resistance in Zambia and other parts of the world [18-21]. During the 2009, 2010 and 2016 cholera outbreaks, Lusaka reported the highest number of cholera cases in Zambia [14]. Therefore, this study sought to describe the phenotypic characteristics of *V. cholerae* O1 strains isolated from Lusaka during these three cholera outbreaks through serotyping and antibiotic susceptibility testing, and their genetic diversity by macrorestriction analysis using Pulsed-Field Gel Electrophoresis (PFGE).

## Methods

**Identification and serotyping of the *V. cholerae* isolates:** isolates used in this study were recovered from rectal swabs and stool samples from suspected cholera cases during the 2009, 2010 and 2016 cholera outbreaks in Lusaka district. After a positive cholera diagnosis by culture, the isolates of *V. cholerae* were stored in skimmed milk tryptone glucose glycerol (STGG) vials at -80°C. The isolates were revived by thawing the vial contents at room temperature and inoculating them in alkaline peptone water at 37°C for 6 hours. They were then subcultured onto Thiosulfate Citrate Bile Salts Sucrose (TCBS) agar and incubated at 37°C for 24 hours. The identity of the isolates was confirmed by biochemical tests as described in the Centers for Infectious Disease Control manual for cholera diagnosis (CDC, 2015) and serology using polyvalent O1 antisera and monovalent Inaba and Ogawa (Mast Diagnostics, Merseyside, United Kingdom) according to manufacturer´s instructions.

**Macrorestriction analysis of the *V. cholerae* isolates:** in order to determine the clonal relationships among the *V. cholerae* isolates, an analysis of chromosomal DNA restriction patterns was performed by Pulsed-Field Gel Electrophoresis (PFGE) with NotI digestion on a Bio-Rad CHEF-DR III electrophoretic system (Bio-Rad Laboratories, Hercules, USA) using a PulseNet protocol. PFGE patterns were analyzed using BioNumerics Version 6.5 Software (Applied Maths, Sint-Martens-Latem, Belgium) with dendrograms of the patterns created using the Unweighted Pair Group Method with Arithmetic Averages, with analysis of banding patterns incorporating the Dice-coefficient at an optimization setting of 1.5% and a position tolerance setting of 1.5%. Two or more isolates with a PFGE pattern percentage similarity value (index) of ≤ 92% were defined as clusters. Strains with pattern similarity value < 92% similarity were each assigned a distinct cluster number. Strains above 95% similarity value were considered identical.

**Antibiotic susceptibility testing of the *V. cholerae* isolates:** antibiotic susceptibility was determined using the Bauer-Kirby disk diffusion test. All strains were tested for resistance to ampicillin (10μg), ciprofloxacin (5μg), tetracycline (30μg), trimethoprim-sulphamethoxazole (25μg) and Nalidixic acid (30μg) using commercially available discs (Oxoid Limited, Hampshire, United Kingdom). Minimum inhibitory concentrations (MIC) of azithromycin was determined using E-test strips (bioMérieux, Marcy I´Etoile, France) according to manufacturer’s instructions. Breakpoints for assessing resistance were determined following the Clinical Laboratory Standards Institute (CLSI) guidelines M45 document [22].

**Ethics approval:** this study was laboratory-based and involved no direct contact with patients. All participant specimens were de-identified and given study-specific identification codes. Permission to conduct the study was sought from the University Teaching Hospital Management in Lusaka, whilst ethics clearance was sought from the University of Zambia Biomedical Research and Ethics Committee (UNZABREC). The ethics clearance certificate number was 014-11-15.

## Results

**Identification and serotyping of the *V. cholerae* isolates:** a total of 83 isolates were successfully revived and were all identified as *V. cholerae* O1. Out of these, 6 isolates were from the 2009 outbreak, while 25 were from the 2010 and 52 from the 2016 outbreaks. The Ogawa serotype accounted for 70% (58), which were from the 2009 and 2016 outbreaks, while the Inaba serotype constituted 30% (25), all of which were from the 2010 outbreak, of the isolates.

**Figure 1:**
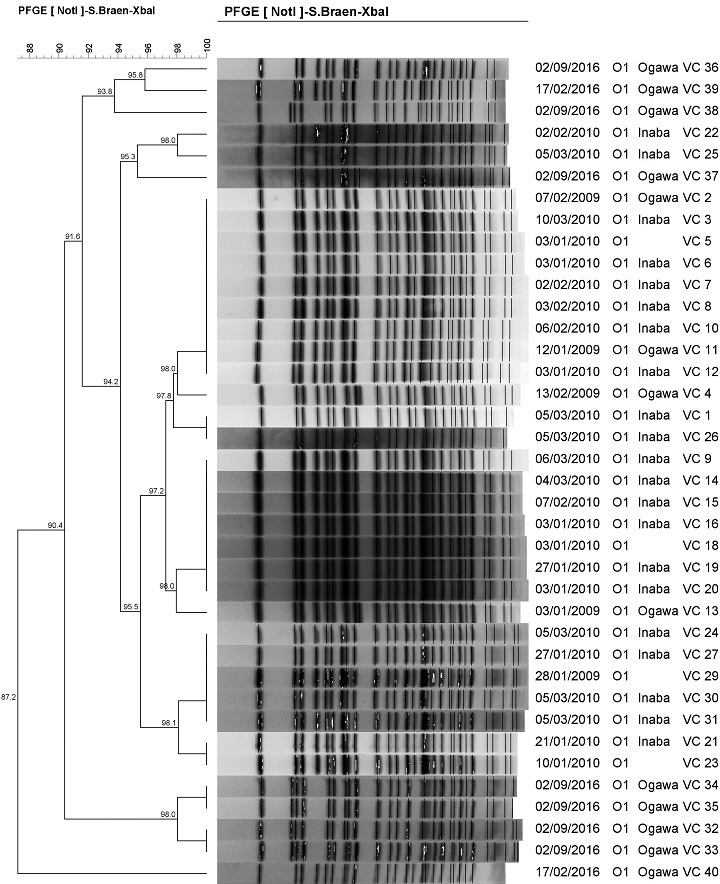
Genetic relationships amongst the PFGE NotI macrorestriction profiles of *V. cholerae* O1 isolates from Lusaka Province

**Macrorestriction analysis of the *V. cholerae* isolates:** PFGE analysis demonstrated high diversity amongst the 38 isolates analysed. When electrophoretic profiles were analysed, 6 clusters, with at least 92% similarity, could be observed (Figure 1). This similarity index (SI) was determined by comparing the three possible cut-offs, 90%, 92%, and 95%. The SI cut-off that provided the best discrimination, after a visual inspection was 92% as it correctly assigned the isolates to their respective clusters. Six clusters were produced with the largest cluster containing (12/38) strains which were 97.8 to 100% similar, followed by a cluster of 8 strains (98 to 100% similarity), then a cluster of 7 strains (98.1 to 100% similarity) and a cluster 4 strains (98 to 100% similarity). The smallest clusters contained 3 strains (93.8 to 95.8% similarity) and (95.3 to 98% similarity). Only one isolate, VC40, remained outside the clusters.

**Antibiotic susceptibility testing of the *V. cholerae* isolates:** all the 2009 and 2010 isolates (31/83) were 100% resistant to nalidixic acid and cotrimoxazole but were all sensitive to ampicillin (100%) and azithromycin (100%). Reduced sensitivity to tetracycline for both the 2009 (67%) and 2010 (64%) isolates was observed, with most of them (65%) falling in the intermediate category. Out of the 52 isolates from the 2016 outbreak, 98% (51/52) were resistant to nalidixic acid. The majority of these isolates were highly sensitive to cotrimoxazole (90%, 47/52), tetracycline (98%, 51/52) and azithromycin (98%, 51/52). Interestingly, reduced sensitivity to ciprofloxacin (6%, 3/52) was observed with most of the isolates (83%, 43/52) falling in the intermediate category for this antibiotic. An attempt was also made to profile antimicrobial resistance (AMR) patterns of the bacterial isolates. All of the outbreaks exhibited AMR but very low multidrug resistance (MDR) was detected. None of the 2009 (0%, 0/6) and the 2010 (0%, 0/25) outbreak isolates were MDR while one of the 2016 outbreak isolates (1.9%, 1/52) displayed an MDR pattern of Tetracycline-Trimethoprim-Sulfamethoxazole-Ampicillin-Azithromycin (Table 1). MDR was defined as resistance to three or more drugs from different classes [23].

**Table 1:** Antimicrobial resistance profile of *V. cholera* O1 isolates from the 2009, 2010 and 2016 cholera outbreaks

Antimicrobial Resistance Patterns	No. of Isolates (%)
**2016**	
TET-SXT-AMP-AZM	1(1.9%)
SXT-NAL-CIP	1(1.9%)
NAL-AMP-CIP	1(1.9%)
NAL-AMP	29(55.8%)
NAL-CIP	1(1.9%)
SXT-NAL	3(5.8%)
NAL	16(30.8%)
Total MDR	1(1.9%)
**2009**	
SXT-NAL	6(100%)
Total MDR	0(0%)
**2010**	
SXT-NAL	25(100%)
Total MDR	0(0%)
Grant Total MDR	1(0.01%)

## Discussion

Cholera outbreaks continue to be major public health threat in Lusaka Province. These outbreaks usually occur in peri-urban townships that are densely populated and have inadequate water and sanitation facilities, a scenario that favours the spread of the disease. The sources of infection have been attributed to contaminated water supplies, contaminated food, inadequate sanitation and poor hygienic practices. Most of these outbreaks occur in the rainy season, thereby predisposing people to the risk of contracting the disease. Data presented in this study demonstrate that *V. cholerae* O1 strains belonging to the Ogawa serotype were responsible for both the 2009 and 2016 outbreaks, while the Inaba serotype was responsible for the 2010 outbreak. Several previous studies have demonstrated that the serotypes Ogawa and Inaba are the most encountered serotypes in other African countries, such as South Africa [2], Tanzania [11], Mozambique [24], Kenya [25] and Ghana [26]. However, a study in the Democratic Republic of Congo (DRC) reported some strains belonging to another serotype, Hikojima, during a 2009 cholera outbreak there [27]. It is interesting to note that a different serotype was reported from the DRC, a country that shares a long border area with Zambia on its southern side. This calls for vigilance against the Hikojima serotype, especially during screening exercises for cholera cases that may arise from this border area. Molecular epidemiology of human bacterial pathogens provides valuable information for understanding the reservoir, pathogenicity and control of bacterial pathogens [28].

Macrorestriction analysis data revealed 16 patterns among all the isolates, with those from 2009 and 2010 outbreak clustering together. This suggests that these isolates were closely related and that those from the 2009 outbreak may have continued to cause infection during the 2010 cholera outbreak. This phenomenon has also been reported in Kenya [25], South Africa [29] and India [30]. The 2016 isolates, however, formed unique clusters, suggesting the emergence of a new clade of the Ogawa serotype causing the cholera outbreak in Lusaka. Similar findings were also reported in a previous Zambian study on outbreak strains isolated between 1996 and 2004 in Zambia, which revealed the emergence of new *V. cholerae* strains [31]. Yet another study in Haiti showed the same phenomenon [32]. These findings suggest that the evolution of *V. cholerae* led to a shift between serotypes Inaba and Ogawa [33]. The strains in this current study generally exhibited high genetic diversity with one isolate, from the 2016 outbreak, not showing any relationship with any of the other isolates. This high genetic diversity seems to suggest that the isolates did not have the same origin [34]. The increase in AMR among enteropathogens, especially MDR strains, is challenging global prospects for fighting diarrhoeal disease pathogens. The emergence and spread of MDR pathogens outstrips the development of drugs, shrinking the therapeutic arsenal [35]. In this study, all the organisms were totally resistant to nalidixic acid and partially resistant to ampicillin. A previous Zambian study, during the 1990-1991 major cholera outbreak, showed low resistance of *V. cholerae* O1 strains to cotrimoxazole, tetracycline, chloramphenicol and doxycycline but significant increase in AMR during subsequent outbreaks [18].

The high resistance observed with cotrimoxazole supports our findings among the 2009 and 2010 isolates, but interestingly very low resistance to this drug was observed among the 2016 isolates. In consonance with our findings from all the three outbreaks, a Nepalese study reported 100% resistance to nalidixic acid [36]. High rates of sensitivity to azithromycin were also reported in a study carried out in Iran, which was in accordance with our findings where only 1.2% resistance was reported [37]. Unlike the findings in this study, high resistance levels to tetracycline (82%) were reported in Mozambique [24]. In this current study, the emergence of MDR strains was observed with the 2016 outbreak where one of the isolates was MDR. These findings were in contrast with the findings of a study conducted in Cameroon where the MDR detection rate was 92% [38]. Strains with new resistance patterns also emerged with reduced sensitivity to ciprofloxacin among the 2016 isolates. V. cholera O1 strains with reduced quinolone sensitivity in Africa were first reported in a Zimbabwean study [39] and later similar findings were reported in Nigeria [40], Kenya [25] and Ghana [26]. Differences in the antibiotic resistance patterns observed during the three outbreaks and the emergence of MDR strains may be attributed to a spontaneous mutation that results from the abuse of antibiotics and horizontal gene transfer [41].

## Conclusion

Data in this study demonstrate that the 2009, 2010 and 2016 cholera outbreaks were caused by *V. cholerae* serogroup O1, with the Ogawa serotype being the predominant serotype. Serotype switching may have occurred in the three outbreaks. However, it is unclear as to what mechanisms could have triggered this process. Another significant finding was that *V. cholerae* strains showed high genetic diversity and therefore, could not have come from a single source of infection. This calls for tracking of the sources of infection as an effective means of controlling cholera in affected areas of Lusaka or other parts of the country. The strains that caused the 2009 cholera outbreak also emerged during the 2010 outbreak but new strains of the bacteria emerged during the 2016 outbreak as evidenced by the macrorestriction and AMR patterns. This study also revealed the emergence of MDR isolates, which were obtained from the 2016 outbreak, with ampicillin and nalidixic acid being the most ineffective drugs. Cotrimoxazole and nalidixic acid were the most ineffective drugs for the 2009 and 2010 isolates. An interesting observation was the reversion to cotrimoxazole sensitivity and reduced quinolone sensitivity for the 2016 isolates. Despite the detection of AMR in all three outbreaks and MDR in the 2016 isolates, cheaper antibiotics such as cotrimoxazole, tetracycline and azithromycin proved to be potent drugs for cholera treatment. This study presents data that underscores the need for close monitoring of *V. cholerae* strains that cause cholera outbreaks in Zambia. This is important to ensure that the correct antibiotic is chosen according to resistance variations, considering the increasing global burden of cholera, and the emergence and spread of new variants that will significantly influence the clinical management of cholera and its prevention.

### What is known about this topic

- Cholera is caused by *Vibrio cholerae* serogroups O1/O139 and serotypes Inaba, Ogawa and Hikojima;
- Emergence of multi-drug resistance has been reported elsewhere;
- Cholera outbreaks in an area can either be caused by highly related or diverse strains.

### What this study adds

- Cholera outbreaks in Lusaka province were due to *Vibrio cholerae* O1 serotype Ogawa in 2009, Inaba in 2010 and Ogawa in 2016;
- Antibiotic resistant strains were circulating in all three outbreaks in Lusaka province but the 2016 outbreak demonstrated emergence of multidrug resistance;
- Macrorestriction analysis demonstrated emergence of new *Vibrio cholerae* variants during the 2016 outbreak in Lusaka province as they formed unique clusters.
